# Mindful parenting—A thematic exploration of narratives from Indian mothers

**DOI:** 10.3389/fgwh.2022.975683

**Published:** 2023-01-11

**Authors:** Ketoki Mazumdar, Sneha Parekh, Isha Sen

**Affiliations:** ^1^ Department of Psychological Sciences, School of Liberal Education, FLAME University, Pune, India; ^2^ National Institute of Mental Health and Neurosciences, Bengaluru, India; ^3^ Independent Researcher, Bangalore, Karnataka, India

**Keywords:** Indian mothers, COVID-19, mindfulness, mindful parenting, parenting stress and wellbeing, qualitative research

## Abstract

**Introduction:**

With the global crisis of COVID-19 continuing, Indian mothers have not received adequate attention with respect to their challenges and mothering experiences. The current study explored mindful parenting practices in a cohort of Indian mothers of children aged 10 years and below that emerged in response to the challenges posed by COVID-19.

**Methods:**

In-depth virtual interviews were conducted with 31 urban Indian mothers to explore their lived experiences of mothering during the global crisis and their engagements with mindful parenting practices. The data were thematically analyzed.

**Results and Discussion:**

The study identified two overarching themes and nine subthemes. The first theme, pandemic-induced stress, included the sub-themes of increased workload, poor support system, lack of time for self, and emotional and physical distress. The second theme of mindful parenting included the sub-themes of awareness as a mother and around the child, acceptance toward self and the child, empathic understanding of self and the child, active engagement with the child, and emotional regulation. Increased workload on all fronts coupled with poor support and a lack of time for self-contributed to exacerbated emotional and physical stress in mothers. They addressed these concerns posed by their lived experiences by engaging in mindful parenting processes in their mothering practices. Mindfulness-based cognitive therapy, mindfulness-based stress reduction, and mindfulness-based parenting techniques could be explored as possible interventions for mothers to alleviate their distress while drawing attention to larger structural changes and policy-level interventions addressing social issues such as gender inequality and childcare concerns.

## 1. Introduction

This paper is an endeavor to understand mindful parenting practices in a cohort of Indian mothers of child(ren) below the age of 10 years that emerged in response to the multifactorial challenges posed by COVID-19. The lived experiences and practices of this particular cohort of Indian mothers along the mindful parenting spectrum to navigate their exacerbated challenges were explored by engaging them in in-depth interviews, which were thematically analyzed to present two large overarching themes of pandemic-induced stress and mindful parenting. This paper discusses the themes along with the nested subthemes in detail with verbatim narrative representations from the mothers to present a slice of their reality to the audience. This paper is one of the first studies to explore the processes of mindful parenting in Indian mothers of young children and thus highlights important issues within the Indian context. Although Indian society reveres a mother's role within the larger sociocultural landscape, their position and identity often get pushed to the sidelines, and their efforts and care work, professional work, and mental work become invisible and unacknowledged. With the ongoing stress of the global pandemic and the deep impressions it has on the gendered nature of mothering, it becomes very pertinent to understand how Indian mothers are coping and navigating these new and old/familiar challenges. There is a definite need to develop a deeper understanding of the process of mindful parenting that has helped mothers to cope with COVID-19 and its associated distresses.

The COVID-19 pandemic is a continuing historical event that has been considered a global crisis posing both physical and mental health concerns. The WHO has declared it a Public Health Emergency of International Concern (1, 2). During the wake of the pandemic, the definition of daily life was given a new meaning—with stringent rules of a common message to “stay at home.” Schools, offices, public places, and daily life transitioned to an online world to adapt to the “new normal.” Along with this new reality came a host of new challenges that families were unfamiliar with, especially the psychological impact of social distancing without access to daily routines and typical activities such as going to school and parks and meeting friends and loved ones. The stress experienced by parents has been documented by researchers to understand the ramifications on their wellbeing, life alterations, poorer quality of relationships, social isolation, and parenting across the world affected by the pandemic (3–8).

### 1.1. Mothering and COVID-19

The pandemic has contributed to unique experiences for mothers, who have been particularly vulnerable with the additional workload of working from home, working for home and attending to their child(ren), taking on homeschooling responsibilities, and ensuring that the home runs smoothly without any additional support (9). Mothers have been doing the bulk of household chores and managing childcare, especially those with young child(ren) in isolation from the day the pandemic was announced for extended periods of time. This daily rigor of engaging in several things together and putting in efforts at multiple shifts in a day for a prolonged pandemic has certainly had implications on mothers’ mental health, including symptoms of anxiety, depression, panic, and exhaustion (10–14), indicating a requirement for support during this critical time.

The pandemic has led to a considerable increase in parenting stress, perceived loss of control, and frequent day-to-day challenges (7, 15–18). Remaining engaged in the active process of mothering with very little support in the forms of domestic help, nannies, schools, and childcare centers under close to near-impossible situations on a daily basis resulted in a depletion of personal resources (10, 19). The majority of urban Indian households, especially where the mother is professionally employed, are found to rely on domestic help for household chores, cooking, and cleaning (20) and on extended family members and childcare centers for their child(ren). According to OECD (21), Indian women spent an average of 536.6 min/day contrasted with an average of 442.3 min/day being spent by men on unpaid care work. Chauhan (22) reported the gender disparities in care work in India with the sudden hike in the overall workload of Indian women due to COVID-19. Globally, women have been spending significantly greater time on unpaid care than men (10, 21) and are disproportionately burdened with care work and mothering (23, 24). COVID-19 has highlighted and deepened this disparity in the gendered nature of a mother's work. This situation potentially impairs a mother's ability to be a supportive caregiver and has been consequently detrimental to their children's wellbeing (7, 10) and has great implications on their physical, mental, and emotional health during the pandemic (9, 25). Considering these factors, there are concerns about how mothers may be coping with the new reality of COVID-19 (14, 22, 26, 27).

Within the Indian context (27, 28), a mother's role, while revered, often displaces her identity into a position of being unacknowledged and invisible (29, 30). Their efforts and associated burdens often go unrecognized. This poses a potential challenge for mothers experiencing additional stress and being unable to cope—all of which have been exacerbated by the onset of the pandemic (22, 26, 27). Taylor (31) called COVID-19 a collective trauma, which is associated with an increase in parenting stress, perceived loss of control, and frequent day-to-day challenges (1, 15, 16, 32, 33).

### 1.2. Mindful parenting

Mindfulness-based practices have been widely researched, and evidence has widely indicated their efficacy in combating stress (34, 35). Kabat-Zinn (36) defined mindfulness as moment-to-moment, nonjudgmental awareness, cultivated by paying attention in the present moment, nonreactively, nonjudgmentally, and as open-heartedly as possible. The principles have been applied across various disciplines, including medicine, neuroscience, healthcare, education, leadership, business, and other major societal institutions, not just limited to psychology (32, 37). Mindfulness-based interventions have been developed for several clinical and nonclinical populations including children (38, 39). Such interventions have also been found to help cope with the increase in stress, therefore building resilience in parents and addressing healthy parent–child relationships, indicating the relevance of incorporating mindful parenting processes into parenting interventions (40–45).

Mindful parenting is defined as “paying attention to your child and your parenting in a particular way: intentionally, here and now, and nonjudgmentally” (44). Several researchers have agreed with the importance of mindful parenting and the benefits of engaging in this process (18, 40, 46–49). Mindfulness-based intervention programs have helped in reducing parental stress and mood disorders (18, 47, 50, 51), boosting parental wellbeing, mindful parenting, and parent–child interaction (43, 52), and improving parenting, coparenting, parental satisfaction, and family functioning (48, 50, 51, 53).

Duncan et al. (43) enlisted five basic mindful parenting characteristics: listening with full attention, nonjudgmental acceptance of self and the child, emotional awareness of self and the child, self-regulation in the parenting relationship, and compassion for self and the child, which aid in promoting more compassionate and sensitive care to the needs of the child. Higher levels of mindfulness in parenting indicated positive experiences of parenting, better parent–child interactions, and lower levels of challenges in parenting compared to a control group (52). Moreira et al. (45) investigated the association between self-compassion and mindful parenting and noted that the process of self-compassion enables parents to be aware of and accept instead of suppressing or disregarding their painful experiences and negative feelings. Such a process aids parents to be aware of and recognize their own emotions in conjunction with their child(ren)'s emotions and experiences, which gives way for parents to self-regulate in their parenting practices through the active process of listening, being there for the child(ren), paying close attention to them, and reflecting more deeply before talking to them (54). Mindful parenting practices could reduce parental stress (55, 56), emotional reactivity, and parental preoccupation with negative parent–child interactions and help in improving self-care, better attunement with the child, and interpersonal functioning (40, 55, 57). Incorporating these mindful parenting practices could help a parent/primary caregiver better manage challenging behavior and situations (41, 58, 59) such as the global COVID-19 pandemic.

Mindfulness as a practice within the Indian population could help mothers improve affect tolerance, enhance flexibility and tranquility, improve focus and mental clarity, and strengthen emotional intelligence and the ability to relate to others and one's self with kindness, acceptance, and compassion. This study hopes to shed light on these needs and the role that mindfulness can play in bettering the lives and experiences of Indian mothers and decreasing the stress they experience while navigating everyday experiences in their role.

### 1.3. Current study

To contribute to empirical evidence in this field of mindful parenting research from the Indian sociocultural landscape, the present study is an endeavor to explore mindful parenting practices in a cohort of Indian mothers to navigate challenges experienced during the COVID-19 period. A thematic exploration has been conducted to better understand pandemic-induced stress and mindful parenting. Previous studies on mindful parenting have followed a quantitative methodology to confirm models or evaluate the effectiveness of mindful parenting interventions (40, 60, 61). Although quantitative methods inform us regarding the effectiveness of mindful parenting, little knowledge has been obtained about how microprocesses and inherent changes take place during the mindful parenting process.

Qualitative research could help to address parents’ direct experiences and provide a more in-depth understanding of previous quantitative results (62). Moreover, a large majority of studies on mindful parenting of mothers were conducted in Western societies, and more research is needed to explore the processes from an Indian sociocultural context, which could further inform parenting interventions to alleviate distress and improve parenting practices. Moreover, the usefulness of such practices within stressful conditions such as the global pandemic also can be highlighted by the current study.

Within the current study, some key constructs include “support,” “time for self,” and “urban Indian mothers.” Support has been defined as any form of assistance provided to the mother or participant from human sources such as another family member, a professional service (e.g., domestic worker), place of employment (e.g., from the management or organization of employment), and systemic support such as through policies and societal norms and acceptance. Time for self has been defined as a period of time that the mother could keep aside purely for herself, that did not involve any other individual and entity, and that could be utilized in a way that the mother or participant liked for herself and the choice was purely hers. Urban Indian mothers have been defined as Indian mothers residing in urban areas (towns or cities) within India. Urban areas are defined as having at least 5,000 inhabitants with a density of 400 people per sq. km or more and at least 75% of the male working population is engaged in nonfarm activities.

## 2. Methods

### 2.1. Participants

The participants of the study were 31 urban Indian mothers with children under the age of 10 years who had completed their higher secondary education and resided in India during COVID-19. The mothers were between 28 and 40 years old. This sample allowed us to explore varied experiences of mothering. Mothers of young children need to actively tend to and care for their children. The mothers were recruited through purposive sampling methods. The study consisted of a cohort of urban mothers from different Indian cities belonging to different cultural backgrounds and family units. Out of the 31 participants, 12 were homemakers (38.71%) and 19 (61.29%) were professionally employed. Regarding family units, 10 (32.26%) belonged to joint family units and 21 (67.74%) belonged to nuclear family units. For this study, a joint family has been operationally defined as a group of people related by blood or through marriage who typically live under one roof and share everyday activities. A nuclear family consists of parents and their children, typically living in one home residence. Twenty-four (77.42%) mothers had one child, while seven (22.58%) mothers had two children at the time of the interview. During the interviews, it was observed that no new themes and subthemes emerged from the 28th interview onward. The researchers felt data saturation was reached, and data collection was stopped after the 31st interview. Please see Table 1 for the socio-demographic profiles of the respondents and Table 2 for a Statistical Summary of the socio-demographic information.

**Table 1:** Sociodemographic information of mothers.

Participant ID	Age (years)	Marital status	Occupation/employment type	Family type	Educational qualification	No. of children
1	36	Married	Part-time	Joint	Graduate	1
2	40	Married	Full time	Nuclear	Doctorate	1
3	40	Married	Homemaker	Nuclear	Doctorate	1
4	34	Married	Homemaker	Joint	Graduate	2
5	33	Married	Homemaker	Nuclear	Postgraduate	1
6	35	Married	Full time	Nuclear	Postgraduate	1
7	33	Married	Full time	Nuclear	Graduate	1
8	34	Married	Part-time	Nuclear	Postgraduate	1
9	30	Married	Full time	Nuclear	Postgraduate	1
10	32	Married	Part-time	Nuclear	Postgraduate	1
11	39	Married	Full time	Nuclear	Postgraduate	1
12	31	Married	Full time	Nuclear	Postgraduate	1
13	35	Married	Full time	Nuclear	Graduate	1
14	38	Married	Full time	Nuclear	Graduate	1
15	36	Married	Homemaker	Joint	Postgraduate	1
16	33	Married	Part-time	Joint	Graduate	2
17	33	Married	Homemaker	Joint	Postgraduate	2
18	32	Married	Part-time	Joint	Graduate	2
19	33	Married	Homemaker	Joint	Postgraduate	1
20	34	Married	Homemaker	Nuclear	Postgraduate	1
21	37	Married	Homemaker	Joint	Postgraduate	1
22	28	Married	Full time	Joint	Graduate	1
23	38	Married	Homemaker	Nuclear	Postgraduate	1
24	30	Married	Homemaker	Nuclear	Graduate	2
25	31	Married	Full time	Joint	Postgraduate	1
26	40	Married	Full time	Nuclear	Doctorate	1
27	36	Married	Full time	Nuclear	Postgraduate	1
28	37	Married	Part-time	Nuclear	Postgraduate	2
29	35	Married	Full time	Nuclear	Graduate	1
30	34	Married	Homemaker	Nuclear	Postgraduate	1
31	33	Married	Homemaker	Nuclear	Graduate	2

**Table 2:** Statistical summary of sociodemographic information of mothers*.*

*N* (mothers)	31
Mean age (years)	34.52
Percentage of mothers engaged in paid employment	61.29
Percentage of mothers living in joint households	32.26
Percentage of mothers with higher education (graduate and above)	100

### 2.2. Procedures

Advertisements for a call for participation in nationwide research were made through social media platforms like Instagram and Facebook. Interested participants contacted the research team's email and were responded to with further details of the study. Appointments for interviews were fixed after receiving written informed consent *via* email, ensuring each participant's comfort with time, virtual space, and technology. Before the interviews started, the participants were informed of the purpose of the interviews and that their anonymized data may be used for research purposes, as mentioned in the consent form. Interviews were conducted one-on-one by a member of the research team. The duration of interviews ranged between 1 and 2 h, depending on the comfort of the mothers to share and hold conversations about their mothering experiences.

Data were collected from October 2020 to May 2021 in the form of telephonic/virtual in-depth unstructured interviews given the restrictions of mobility in India during COVID-19. No incentives were offered to the respondents for participating in the study. The interviews were conducted in English and recorded and transcribed as soon as the interview got over to enable the researchers to include memos and annotations from their field notes to the transcribed interview. Moreover, the interviews were transcribed orthographically to retain the true essence of the data by the inclusion of utterances such as “umms, y’all, y’know, ah” as well as noting their pauses and exclamations. The participants’ names, locations, and other identifying details were omitted and/or pseudonymized to ensure confidentiality. The lines of inquiry were open-ended, guided by an interview schedule exploring the mothering experiences within the pandemic context—focusing on their challenges and potentially helpful strategies. The interview questions were phrased in a way that allowed participants to ask for any clarification if needed. Some sample questions from the interview schedule included the following: “During the lockdown, have you noticed any changes in your parenting practice? If yes, please elaborate?”, “Explain what happens when things don’t go as planned?”, “How did you process your thoughts and emotions when going through a difficult day?”, “Describe any changes that you have noticed in your mothering since the lockdown?”, “How do you respond to your child when they are being impatient in the middle of a working day?”

### 2.3. Analytical plan

All three researchers engaged in-depth with data collection and analysis to ensure data trustworthiness. The credibility of data was achieved through investigator triangulation, whereby three researchers were engaged in the process of coding, analysis, and interpretation. To ensure the interpretative rigor of the coding process, an intercoder agreement was utilized to ensure coding accuracy and monitor intercoder reliability among the research team throughout the analysis stage. This was done through regular meetings of team members holding different perspectives toward reaching a consensus in interpreting and analyzing data. The differing research backgrounds and training orientations of the researchers were also kept in mind throughout the analytic process. By questioning the influence of these pre-existing contexts, the researchers tried to build the analysis strictly from the data. All of these steps balanced out the potential bias of individual researchers and aided in maintaining the credibility and confirmability of data.

The intercoder agreement was established using the following protocol:

(1) Two team members and the principal investigator coded all interviews separately.
(2) Each researcher made their interpretations and wrote down key emerging concepts.
(3) At each analytic stage, discussions were held to resolve any coding and conceptualizing incongruities and reach a consensus. No significant edits were made.
(4) The themes and emerging concepts were maintained in a codebook attached at the end of the paper.
(5) During the whole process, memos were written to make a note of ideas that were used to develop concepts and codes, which helped direct the analytic procedure.

The data were thematically analyzed and interpreted following a guide introduced by Braun and Clarke (62), which outlines a six-step procedure toward thematic analysis. Thematic analysis was considered an appropriate analytic technique as this study intended to better understand the views and experiences (63) of Indian mothers in the context of the pandemic. Furthermore, it is epistemologically and theoretically flexible and independent (62). The audio-recorded interviews were transcribed verbatim before starting the process of thematically analyzing them. The researchers acquainted themselves with the data and studied the transcripts to look for and note down the initial ideas. The second phase involved the researchers systematically producing as many initial codes from the respondents’ data as possible. The third phase started with the different codes being sorted into potential themes, retaining all major themes and subthemes from the data, while the fourth phase consisted of identifying the themes and checking their association with the coded extracts and the entire data set. In the fifth phase, refining the specifics of each theme along with clear definitions of and concise names for the themes was developed. The interviews were analyzed inductively (64) to allow this process to develop concepts and constructs from the data without any predetermined theoretical framework in mind. A repetition of themes was noted during analysis, and participants’ mothering experiences could be coded within a common or shared set of themes and subthemes. In the last phase, the report was produced with the final analysis of the selected extracts. After the initial coding process, the codes were examined for similarities or differences. Transcripts were analyzed individually, and emerging themes were noted. Comprehensive themes were generated based on this analysis across all transcripts.

Quotations from the respondents’ narratives were used to ensure that their voices are adequately represented. The authors selected the particular quotes to represent the findings to extend upon and bring to life the point they were making at that juncture in the narrative, such that the intended meaning gets augmented. This was achieved through making strategic choices to select the specific verbatim to effectively convey the illustrative message that was appropriate for the particular finding in the manuscript. In the process of choosing wisely the represented quotations, the authors attempted to capture a window into the human story that led the researchers to “know” something in a new way. Using quotations wisely brings the complex theoretical basis under consideration into everyday relief, helping the reader move back and forth between generalized observations and distinctly particular experiences of them to gain the kind of robust insight into the research conducted.

## 3. Results

The following section will discuss the themes that emerged from the data in light of the relevant literature. Two themes were identified—pandemic-induced stress and mindful parenting along with nine subthemes. Four subthemes identified under pandemic-induced stress included increased workload, poor support system, lack of time for self, and emotional and physical distress. Mindful parenting included the subthemes of awareness as a mother and around the child, acceptance toward self and the child, empathic understanding of self and the child, active engagement with the child, and emotional regulation. The narratives were indicative of the challenges and the potential helpful strategies for the cohort of Indian mothers. Moreover, they also reflected on how the mothers became more mindful of themselves and their interactions with their child(ren) during the pandemic. The following section will take the reader through the findings of the study. Please see Appendix 1 for the Codebook.

### 3.1. Pandemic-induced stress

The narratives of mothers were laden with stressful experiences of mothering amidst COVID-19, reflecting the challenges they faced. The following section will present the subthemes under the pandemic-induced stress theme and give the reader an opportunity to understand their lived experiences, which will be supported by voices in the form of quotations from the mothers.

#### 3.1.1. Increased workload

The first subtheme highlights the increased workload for mothers such as that of increased childcare, homeschooling, and professional and amplified household responsibilities. Rina, a 30-year-old mother to a 5-year-old child, spoke about her daily schedule and how she had to multitask to fulfill her household and professional responsibilities, “*By the time it was 6:00 or 6:30 p.m. I used to be free from all the work, and again I used to sit for meetings and respond to emails. In between while having lunch, I have attended meetings and even washed utensils on certain busy days in the middle of work!*” Further, with everybody locked-up at home along with working from home, there were additional difficulties in navigating the multiple roles of a mother effectively. Tanya, a 32-year-old homemaker and a mother to a 7-year-old child, reflected on her experiences of homeschooling her daughter and other necessities that landed squarely on her shoulder, “*She is a toddler, and her classes are more like my classes actually. So, she's not attending, I am attending, I have to be present throughout her classes and also ensure all household chores get done, food gets cooked, everybody gets fed!*” A significant addition to their existing extensive professional and household workload was making their child(ren) transition to and attend online school. Mothers had to ensure the children were ready for school, remained attentive, and completed their homework all within the four walls of their house, often balancing other tasks of running the household smoothly, attending office (if professionally employed), and tending to other members of the family, without much external support. Sabrina, 40-year-old, was mother to a 6-year-old, elaborated on her situation to elucidate this phenomenon, “*So at 10:00, once his class starts, we are in the same room, in the background the teacher and children are talking, and I need to have one eye on him and another on my office laptop responding to my boss and team; basically, multitasking on a different level*.”

#### 3.1.2. Poor support received by the mothers

Contributing to the challenges of increased workload was the poor support received by the mothers, which was identified as the next subtheme. Narratives of the mothers were indicative of the absence of house help, poor support from family members, and lack of acknowledgment during the global crises. Jaya, a 35-year-old Chartered Accountant and mother to an 8-year-old, explained, “*my house help would do everything from cleaning to cooking and was a huge source of support. So, a four-hour job that was outsourced was put back on my shoulders all of a sudden*.” Jaya's words exemplified how urban and semiurban mothers relied on outsourced help to take care of many household chores, which they were bereft of at the strike of the pandemic and social isolation. In addition, the respondents elaborated on how some support from their husbands or in-laws could have helped ease their exacerbated workload. Ira, a 34-year-old lawyer and mother to a 1-year-old child, shared, “*when I was almost getting into postpartum depression in the midst of lockdown, to be honest, that is when my mother-in-law asked one of her live-in house help to help me look after Aryan and asked me to seek professional help from my ob-gyn. And I think that if that help was offered earlier, it would have been much helpful*.” Aanya, a 31-year-old mother to a 2-year-old child, also spoke about the lack of acknowledgment around responsibilities associated with childcare, which made her mothering efforts invisible, “*there is a lot of hard work behind a baby. He felt that I was doing nothing, that I am just feeding her and helping do the housework. All that hard work, yet nothing is acknowledged*.”

#### 3.1.3. Lack of time for self

The above experiences contributed to the cohort of mothers having less time for themselves, which is explored in the next subtheme, lack of time for self, where the mothers explored their lack of me-time, lack of self-care, and disturbed routine during the pandemic. “*I worked out, used to have a routine, but now it's hard for me to sleep on time, wake up on time, take out some time to work out. These things are not possible now, so frankly I don’t like it*.” Luna, a 33-year-old mother to a 7-year-old child, expressed her frustration. Mothers did not find time to engage in self-care or even have some time to relax. Reena, a 37-year-old mother to one 5-year-old and one 1.5-year-old child, explained how even finding an hour for herself was difficult, “*I want a me time, kid free, office-free, household chores-free, me-time, that is what I dream of every single day, one hour just for myself*.” Managing multiple roles and activities throughout the day led to a complete disruption of their daily routines. Vini, a 37-year-old mother to a 3-year-old, explained, “*With my husband working from home and my toddler attending her classes online, it was utterly chaotic because you know it's like everybody needs me all the time. It's like I have to rush through the day, my whole routine has gone for a toss*.”

#### 3.1.4. Emotional and physical distress

Being actively engaged in mothering along with the daily rigor of managing multiple things in multiple shifts for a prolonged pandemic, in the absence of or little support in the forms of domestic help, nannies, schools, childcare centers, resulted in a depletion of personal resources and had implications on their mental wellbeing. This experience is explored in the next subtheme, emotional and physical distress, which was a detrimental consequence of the pandemic on the mothers. For new mothers, it was particularly challenging as they found no recourse and support to manage their stress and worry. Alia, a 28-year-old mother to a 1.5-year-old child, expressed, “*The postpartum phase is difficult. There were so many changes and there were times I used to miss my mom but I couldn't go to her because of the social distancing and lockdown. I used to cry in the shower*.” Children, especially younger, also unwittingly add to the pressures mothers experience by seeking attention. Meeting their needs can be stressful to mothers in the absence of multiple avenues of proactive engagements such as schools, parks, co-curricular activities and support in the form of extended family members, nannies, creches, and entertainment such as stepping outside the home for social gatherings. Nikita, a 40-year-old mother to a 9-year-old child, shared, “*Sometimes it so happens that my focus is just on one thing and in such situations when she would come and say ‘Mama I have finished my work, now what should I do?’ It would take a minute to think of what to tell her and I would feel angry at the interruption and all the different things I have to take care of*.” The circumstances of the pandemic were exceptionally overwhelming, with mothers unable to regulate their emotions and finding it tough to manage their new routines. This sentiment was highlighted by Shreya, a 34-year-old mother to a 6-year-old child “*I used to cry, thinking of the fact that how do I manage all of this on my own?*” Along with experiencing emotional distress, mothers’ physical health also took a toll, with many encountering sleep deprivation and severe lack of rest. This is evident through Sia's, a 30-year-old mother to two children, aged 3 and 7 years, narrative, *“…It took a toll on my sleep also. After a month and a half, I told my husband, this is not working out and I actually broke down in front of him that I am very tired, I am sleepless. You have to pitch in*.” These physical travails intermingled with the emotional anguish and amplified the overall distress that the mothers felt. Without much support and tremendous change in the internal and external environment due to the pandemic, mothers experienced significant emotional and physical strains, especially when they were required to balance everything without much support or resources, and this could potentially have an impact on their caregiving abilities and being mindfully aware of their child(ren)'s and their own needs.

### 3.2. Mindful parenting

Living through the various changes and uncertainties faced during COVID, mothers attempted to adapt and cope by being more mindful. This led to the second theme of mindful parenting.

#### 3.2.1. Awareness as a mother and around child

The first subtheme explores awareness as a mother and around the child, as reported by the respondents. Mothers became more aware of their own emotions, behavior, and limitations and also those of their child(ren). Sabrina, a 40-year-old mother to a 4-year-old child, spoke about how the difficulties of working from home due to the pandemic impacted her emotions and parenting responses, “*I have to close the door when I take my classes so that I do not get interrupted by my daughter, yet there have been so many times, she comes banging in and asks for a toy or a question that she seeks an answer for at that instant! I get angry but then I realise I am all she has!*.” Ruchi, a 36-year-old mother to a 5-year-old child, further expressed concerns about her child's development with respect to his behavior and engagement, “*he started enjoying the lockdown, because he was getting access to unlimited television, he was getting mobile to play with especially when I was in a meeting or cooking, he wasn't getting enough outdoor playtime. All that is not good for his emotional and cognitive development and he doesn't understand that*.” Working from home gave Elina, a 33-year-old mother to a 6-year-old child, the opportunity to observe her child's behavior more closely. She went on to say, “*I realised that her behaviour was beginning to get a little violent after watching some cartoons and I started becoming more aware and present during the day when she was playing. Yes, it was tough to balance my office work and be a hands-on mother, but I had to do it for the benefit of my child*.” The close observation allowed her to understand, reflect on, and thereby identify the reasons for behavioral change.

#### 3.2.2. Acceptance toward self and the child

The narratives of the mothers also reflected acceptance toward self and the child, which forms the next subtheme of mindful parenting. Nina, a 35-year mother to a 3.5-year-old child, shared, “*I had written on the calendar that this day, he should learn letter A or he should do this. And I realized it just adds to my frustration because there's no way your child is going to go by your time and schedule. He has his own timetable*.” It was acceptance of her child's limits that allowed Nina to deal with the distress of her plan or goals not being achieved as expected. She could give him the space and time he required to learn. Tina, a 33-year-old mother to a 5-year-old child, elaborated Nina's viewpoint by sharing, “*for a 5-year-old, something is being taught on the other side of the laptop where he doesn’t know who the teacher is, who his classmates are and we expect him to learn something, this is a wrong kind of expectation*.” As is evident in this quote, mothers became more open and understanding of their children's predicament and learned to work around the child's needs as opposed to imposing their own schedule and needs on the child. Mothers in the study spoke not only about being accepting their children but also their own emotions and limitations. Maya, a 34-year-old mother to two children, aged 4 and 8 years, shared, “*at times if it is a very emotional kind of an experience, I do end up feeling bad, feeling a little annoyed at times but then as I told you I have come to believe that you have to be in the present moment and accept it*.”

#### 3.2.3. Empathetic understanding of self and the child

Awareness and acceptance allowed mothers to have an empathic understanding, which is discussed under the next subtheme of empathetic understanding of self and the child. Mothers realized that their children were equally stressed due to the pandemic and expressed empathy for the children and themselves as mothers. Lisa, a 36-year-old mother to a 2-year-old child, shared, “*Sometimes I would feel angry. What all should I take care of? But then I would realize she is a child, we ‘should’ understand. Yet, sometimes I feel frustrated*.” She felt empathy for herself and her child in the given situation. Kavya, a 33-year-old mother to two children, aged 5 and 7 years, reflected, “*when lockdown started getting extended children also began becoming impatient because they were not able to go to school, they were not able to meet their friends. We were looking after their physical activities, whatever at home we could do but they were not able to go out and meet their friends which is a very important part of growing up*.” Understanding the situations from their child's point of view allowed mothers to show compassion toward them. Meena, a 35-year-old mother to two children, aged 3 and 5 years, could understand her children's unexpressed needs and experiences, “*They are so small, that they are neither able to tell properly nor understand. But, the memories of this time are definitely going to stay with them for long so I try to make their days filled with learning, reading and new games and plays; We have family board game nights every weekend and this has certainly made our bonding stronger*.” An empathic stance toward their children allowed mothers to show compassion and make their best attempts to increase their positive experiences during the challenging pandemic times.

#### 3.2.4. Active engagement with the child

Being aware, accepting, and empathetic toward their children allowed mothers to see their needs more clearly. Mothers chose to actively engage with their children, which forms the fourth subtheme of active engagement with the child. Shruti, a 38-year-old mother to a 6-year-old child, mentioned that she developed a new way of engaging with her child during the lockdown, which involves conversation-based activities, “*Even if I get 15–20 minutes, we spend that time talking to each other in the morning; after my exercise we sit with a cup of tea and milk in the balcony, before our online world beckons us. It is just our talking time, seeing the birds, observing this and that, engaging in whatever conversation she wants to have. I love those conversations with her, this is something new that we have introduced during the lockdown*.” Jaya, a 36-year-old mother to a 6-year-old child, spoke about listening to her child with undivided attention, “*I sit with her and listen to her complain about her not being able to meet her friends, go to park to play, meet her grandparents, even if it goes on for an hour and I would empathize with her*.” Attentive listening emerged as one of the components of interaction.

#### 3.2.5. Emotional regulation

Although mothers also experienced stress due to the pandemic and lockdown, narratives were suggestive of the choice mothers made to work on their emotional expressions, which formed the fifth subtheme of emotional regulation. Jaya, a 36-year-old mother to a 6-year child, shared, “*I sometimes keep my mouth shut and move away from the situation so as not to have an emotional outburst*.” Aanya, a 31-year-old mother to a 5-year-old child, explained how she controlled her expressions as well, “*And then I try to calm myself down by counting backwards or I tell my husband, please look after our child so that I can pacify myself*.” Meena, a 31-year-old mother to a 7-year-old child, could identify how a change in her behavior impacted her child, “*I have become patient with her so she has also become patient with me and her family members*.” Mothers in the study showed the ability to work on their emotional expressions at the moment for the benefit of their children. Nadia, a 39-year-old mother to a 6-year-old child, further shared, “*I want my daughter to be able to accept all her emotions as they are, and be aware of what each emotion does to us. I also simply want her to be able to express freely however she is feeling. That's very important so over time both of us have learnt to manage our emotions and I want her to learn to identify others’ emotions as well*.” She could better understand her child's emotions and wants her child to develop the same skill in understanding others’ states of mind.

## 4. Discussion

As one of the first studies to explore the processes of mindful parenting in Indian mothers of young children, this study sheds light on some important issues. It is clear that there is a need to better comprehend their lived experiences toward engaging in mindful parenting processes to cope with COVID-19 and its distress. This information could also help generate more interventions focused on alleviating stress associated with mothering at large. Mothers realized quite early on in the lockdown how staying indoors impacted both their mothering practices and their children's response to them. Changes such as not being able to go out to play, attend school, and meet their friends and staying in homes with busy parents culminated in children being glued to either screens or having emotional outbursts.

The cohort of mothers from the study reported experiencing an increased workload on all fronts during the pandemic, especially increased household work, professional work, and childcare. Similar findings have been reported by several studies worldwide (9, 22, 24), where they reported how the pandemic was associated with increased parenting stress, perceived loss of control, and frequent day-to-day challenges (7, 15–17, 33). The multiple responsibilities that a mother had to start paying heed to on top of their already numerous lists of things to do led to increased domestic conflicts as a result of homeschooling (65), and this further added to the overall burden during COVID-19 (66, 67). With an increase in workload, mothers also shared the lack of support they received from all fronts, starting with their domestic helpers whom they have depended on for a long time. Indian households, especially in the urban and semiurban domiciles, rely on outsourced help in their daily activities (20). In the absence of house help for cleaning, cooking, and other daily chores, mothers had to take up additional responsibilities (29, 68), as also indicated in the narratives of the current interviewees. This was intensified by poor support from other family members (68). Globally, women are disproportionately burdened with care work and mothering (23, 24), and COVID-19 has further intensified this gender role disparity (22).

Studies have reported on the mental health and physical health implications of the heightened work and stress during COVID-19 (10, 12–14, 19, 22), and this was observed in the reflections of the mothers from the study. Previous studies have also found the negative impact of the lockdown and social distancing restrictions during COVID-19 on a mother's postpartum mental wellbeing (69–72), as discussed by a mother who had a newborn child to take care of in the middle of the pandemic, staying away from her family and support systems. Mothers’ experiences of overwhelm and strain were consistent with studies conducted worldwide on mothers and parents during the pandemic (7, 10, 14, 22, 33, 70, 72, 73)*.* Prolonged periods of stress were found to be associated with cognitive, emotional, and physical exhaustion (74). Along with the experiences of mental stress, studies have also reported how the pandemic has caused physical stress on mothers. This type of reality has been reported to put parents at a higher risk of experiencing distress and impinging on their caregiving abilities (7, 10, 22).

Although mothers were also distressed during this period, they were dedicated to their mothering duties. Instead of getting caught in the spiral of stressful situations, they chose to parent mindfully, as observed in their narratives. Kabat-Zinn and Kabat-Zinn (44) defined mindfulness as moment-to-moment, nonjudgmental awareness, cultivated by paying attention in the present moment, nonreactively, nonjudgmentally, and as open-heartedly as possible. Mindful parenting is the application of principles of mindfulness to parenting (43). Duncan et al (43). identified emotional awareness of self and the child as a characteristic of mindful parenting. This was synonymous with the first subtheme that emerged under the broad theme of mindful parenting; awareness of self as a mother and around the child. Mothers spoke about being more aware of their own emotions, behavior, and limitations and those of their child(ren). The current study identified that the period of lockdown was associated with multiple difficulties; it also provided mothers the opportunity to be with their children more, thereby allowing for closer observation. Mothers expressed concern over the impact of the pandemic on their children while acknowledging their own emotions. Narratives in the study were reflective of acceptance toward emotions and limitations of oneself and those of the child(ren). Nonjudgmental acceptance toward one's emotional experiences has been shown to be a factor in parental wellbeing and children's internalizing behaviors (40, 42, 57). Kabat-Zinn and Kabat-Zinn (44) identified paying attention nonjudgmentally as a component of mindful parenting. This is found to improve the effectiveness of parenting interventions (42, 45). A similar theme was identified by Duncan et al. (43); nonjudgmental acceptance of self and the child.

Despite the ill effects of the pandemic faced by the mothers, emotionally and with respect to the constraint of time for self-care, narratives were indicative of mothers being empathic toward their child(ren) and engaging more actively with them. Mothers spoke about how they began giving time to listen to their child(ren), allowing them to express themselves. The narratives were found to be synonymous with listening with full attention, one of the characteristics of mindful parenting (43). Mothers expressed concern over their children being equally stressed and losing out on opportunities they would have normally had. Empathic understanding toward children emerged as a subtheme under the broad theme of mindful parenting. Compassion for the child(ren) is found to be associated with positive affect and support toward children (43). It also allows parents to recognize their own and their child(ren)'s emotions and experiences, allowing them to regulate their parenting practices (54). Narratives were indicative of mothers actively engaging by being aware of their own emotions at the moment and regulating it for the benefit of their children. These findings are synonymous with self-regulation (45, 52, 54, 55), one of the characteristics of mindful parenting (43). Mindful parenting has been found to help parents be fully present during interactions (43) and be more aware of their communication with children while being more aware of their parenting stress and regulating it, which may improve their emotional availability (75).

The current researchers cannot help but notice how awareness toward self and their children may have been the key to experiencing or practicing the other components of mindful parenting, such as acceptance and quality of engagement. Higher awareness allowed the mothers to understand their child(ren) from their perspective rather than their own, allowing them to choose to spend uninterrupted time with them despite the lack of time for themselves to rest and work on their emotional expressions despite their own emotional distress.

Research has found that isolation and increased workload impair a mother's ability toward caregiving, which could be detrimental to the wellbeing of children (7, 8, 10, 15, 33, 65, 67, 70). Children required a lot more engagement during the pandemic, which often took a toll on mothers both physically and emotionally. Higher mindfulness in parenting was found to be associated with a higher level of positive parenting and improved communication with their children (43), which were also identified in the narratives as active mothering processes. Responding to the demands of the situation, the mothers who participated in the study reported adopting mindful parenting approaches as a means to navigate the global crises.

The findings clearly indicate that the mothers chose to parent mindfully with respect to the challenges they faced as a mother. However, it would be interesting to explore what factors helped them choose to parent mindfully and to give time to their children despite the paucity of time for themselves.

## 5. Conclusion

The COVID-19 pandemic has been considered to be a global crisis that has impacted parents and mothers mainly due to the additional workload of working from and for home, attending to their child(ren), taking on homeschooling responsibilities, and ensuring that the home runs smoothly without any additional support. Findings from the study indicate that increased household work, professional work, and childcare responsibilities along with adapting to online schooling contributed to an overall increase in workload for mothers. Disturbed routine due to multitasking and managing several roles deprived mothers of time for themselves, and they could often not care for themselves. These along with the absence of house help, poor support, and a lack of acknowledgment from family members exacerbated emotional and physical distress. Mothers also reported a lack of acknowledgment or how their mothering efforts were rendered invisible. Instead of succumbing to the stressors of the pandemic, mothers dedicated to their role adopted a mindful approach to their parenting. Mothers became aware and accepting of themselves as a mother and of their child(ren). They could also empathize with themselves as a mother and with their children while engaging in active mothering. Mindful parenting is an intricately linked process that plays a role in one's perceptions and response toward self and helps improve the parenting journey and therefore interactions with the child(ren). In particular, these processes became even more crucial to tide over the uncertain and challenging phase of the COVID-19 pandemic.

The experiences of the mothers in this study resonate with some of the experiences of mothers seen globally, as highlighted in the existing literature (47, 52, 55). Those practicing mindful parenting have been seen to have higher levels of mindfulness and self-compassion traits. They are also seen to experience less stress. This is strong evidence for using mindfulness principles and applying them to mental health interventions for mothers across regions and ethnicities.

### 5.1. Limitations

The participants in this study were predominantly from a middle and upper-middle economic stratum and based in urban settings in India. This limits the generalizability of the findings to other geographically and economically diverse community settings. This highlights the need to capture the lived experiences of a more ethnically and socioeconomically diverse group of mothers to whom India is home. The sample also consisted of nonclinical mothers, which limits the applicability of the findings to clinical and community samples. The participants’ lived experiences were also examined within a very specific context of COVID-19.

### 5.2. Implications and future research

This study adds to the limited empirical evidence on mothering experiences in India. A nuanced understanding of the role of mindful parenting practices may be relevant to develop psychological interventions and support mothers in the context of the pandemic, especially if more waves of the virus and lockdown curtailments are expected in the future. Mindfulness-based cognitive therapy, mindfulness-based stress reduction, and mindfulness-based parenting techniques could be explored further in the Indian context as possible psychotherapeutic interventions to improve parenting and reduce parenting stress.

These results also have implications for understanding the long-term effects of COVID-19 on maternal mental health. Since the pandemic shares key features with that of a highly stressful situation, having negative implications for mother–child relationship, the knowledge developed as part of this research can be applied beyond COVID-19 as well. Mindfulness when practiced over time is understood to develop as an adaptive trait. The skills developed during the pandemic are not context-specific. These are expected to become a part of the mother's skill set and therefore be generalized beyond the pandemic to day-to-day reflections and interactions. Future studies may explore whether and how skills developed during the pandemic were generalized to postpandemic everyday living. Future studies could specifically examine mothers within a rural and lower socioeconomic stratum sample to understand the similarities and differences for this group. Cross-cultural studies among mothers compared to other Asian cultures and Western nations would add valuable data to the existing literature. Additionally, COVID-19 had laid bare many long-standing disparities and stressors that have the opportunity to be changed. Thus, future research can also focus on calling for structural-level changes and policy-level interventions addressing social issues such as gender inequality and childcare concerns.

## Data Availability

The raw data supporting the conclusions of this article will be made available by the authors, without undue reservation.

## Appendix 1. Codebook for major themes, subthemes, and codes.

**Table:**

Themes	Subthemes	Codes
Pandemic-induced stress	Increased workload	Increased household work, professional work, and childcare
	Poor support received by mothers	Absence of house help, lack of support from family members, and lack of acknowledgment
	Lack of time for self	Lack of me-time, lack of self-care, and disturbed routine
	Emotional and physical distress	Experiencing heightened emotionality, physical stress, and an increase in physical and emotional work
Mindful parenting	Awareness as mother and around the child	Awareness of limitations, emotions and behavior of oneself as a mother and of the child(ren)
	Acceptance toward self and the child	Acceptance towards limitations, emotions and behavior of oneself as a mother and of the child(ren)
	Empathic understanding of self and the child	Neutral attitude toward shortcomings as a mother and care for the child(ren)
	Active engagement with the child	Undivided attention while listening and conversation-based activities
	Emotional regulation	Controlling expression of negative emotions toward the child(ren) and emotional self-checks
